# Long-term safety and tolerability of atabecestat (JNJ-54861911), an oral BACE1 inhibitor, in early Alzheimer’s disease spectrum patients: a randomized, double-blind, placebo-controlled study and a two-period extension study

**DOI:** 10.1186/s13195-020-00614-5

**Published:** 2020-05-14

**Authors:** Gerald Novak, Johannes Rolf Streffer, Maarten Timmers, David Henley, H. Robert Brashear, Jennifer Bogert, Alberto Russu, Luc Janssens, Ina Tesseur, Luc Tritsmans, Luc Van Nueten, Sebastiaan Engelborghs

**Affiliations:** 1 Janssen Research and Development LLC, 1125 Trenton-Harbourton Rd, Titusville, NJ 08560 USA; 2 Janssen Research and Development, a Division of Janssen Pharmaceutica NV, Beerse, Belgium; 3 Reference Center for Biological Markers of Dementia (BIODEM), Institute Born-Bunge, University of Antwerp, Antwerp, Belgium; 4 Present address: UCB Biopharma SPRL, Chemin du Foriest, B-1420 Braine-l’Alleud, Belgium; 5 Janssen Research and Development LLC, Raritan, NJ USA; 6 Department of Neurology and Center for Neurosciences, UZ Brussel and Vrije Universiteit Brussel (VUB), Brussels, Belgium

**Keywords:** Atabecestat, BACE1 inhibitor, Alzheimer’s disease, Predementia, Amyloid, Aβ, Cognition, Liver enzyme elevation

## Abstract

**Background:**

Atabecestat, a potent brain-penetrable inhibitor of BACE1 activity that reduces CSF amyloid beta (Aβ), was developed for oral treatment for Alzheimer’s disease (AD).

The long-term safety and effect of atabecestat on cognitive performance in participants with predementia AD in two phase 2 studies were assessed.

**Methods:**

In the placebo-controlled double-blind parent ALZ2002 study, participants aged 50 to 85 years were randomized (1:1:1) to placebo or atabecestat 10 or 50 mg once daily (later reduced to 5 and 25 mg) for 6 months. Participants entered ALZ2004, a 12-month treatment extension with placebo or atabecestat 10 or 25 mg, followed by an open-label phase. Safety, changes in CSF biomarker levels, brain volume, and effects on cognitive performance were assessed.

**Results:**

Of 114 participants randomized in ALZ2002, 99 (87%) completed, 90 entered the ALZ2004 double-blind phase, and 77 progressed to the open-label phase. CSF Aβ fragments and sAPPβ were reduced dose-proportionately. Decreases in whole brain and hippocampal volumes were greater in participants with mild cognitive impairment (MCI) due to AD than in preclinical AD, but were not affected by treatment. In ALZ2004, change from baseline in RBANS trended toward worse scores for atabecestat versus placebo. Elevated liver enzyme adverse events reported in 12 participants on atabecestat resulted in dosage modification and increased frequency of safety monitoring. Treatment discontinuation normalized ALT or AST in all except one with pretreatment elevation, which remained mildly elevated. No case met ALT/AST > 3× ULN and total bilirubin > 2× ULN (Hy’s law).

**Conclusion:**

Atabecestat was associated with trend toward declines in cognition, and elevation of liver enzymes.

**Trial registration:**

ALZ2002: ClinicalTrials.gov, NCT02260674, registered October 9, 2014; ALZ2004: ClinicalTrials.gov, NCT02406027, registered April 1, 2015.

## Background

Cleavage of the amyloid precursor protein (APP) by β-secretase1 (BACE1) is the first and rate limiting step in the production of amyloid beta (Aβ), the main constituent of amyloid plaques, a hallmark pathological feature of Alzheimer’s disease (AD). Subsequent cleavage by γ-secretase results in the production of Aβ_1–42_, which has a high tendency to aggregate, as well as other Aβ fragments of shorter length, of which Aβ_1–40_ is the most prevalent. These Aβ forms can also aggregate to form oligomers and fibrils [1, 2]. Thus, inhibitors of BACE1 prevent the formation of Aβ_1–42_ as well as Aβ_1–40_, Aβ_1–38_, and Aβ_1–37_ and are potentially disease-modifying therapeutic agents in the treatment of AD.

Supporting this mechanism is the observed correlation between the catalytic efficiency of BACE1 for its substrate APP and the occurrence of AD. The Swedish APP mutant (KM670/671NL), a more efficient substrate for BACE1 (10×), causes a rare familial form of AD that is inherited in the dominant Mendelian fashion. On the other end of the spectrum is an allelic variant of APP (A673T), a less efficient substrate for BACE1 (0.5×), which is protective against sporadic AD [3].

Atabecestat (formerly JNJ-54861911) is a potent brain-penetrable BACE1 inhibitor developed by Janssen Research & Development in collaboration with Shionogi as an oral treatment of AD, with reduction of CSF Aβ as its primary mode of action [4]. In phase 1 studies in elderly Caucasian and Japanese participants with preclinical AD and mild cognitive impairment (MCI) due to AD, treatment with atabecestat at 10 mg and 50 mg for 4 weeks significantly reduced from baseline CSF levels of Aβ_1–40_ by 67% and 90%, respectively, as compared to placebo and was well tolerated [5]. Similar reductions were observed in all other Aβ fragments. The observed steady-state CSF Aβ_1–40_ reductions from baseline were within the 95% confidence interval predicted by pharmacokinetic/pharmacodynamic modeling from a multiple ascending dose phase 1 study in healthy population (5 to 50 mg once daily for 14 days).

We report two phase 2 studies (ALZ2002 and ALZ2004) whose primary objectives were to assess the long-term safety and tolerability of atabecestat in patients with early AD (predementia AD; encompasses preclinical AD to mild cognitive impairment [MCI]). The parent study, ALZ2002, was a randomized double-blind, placebo-controlled study designed to investigate the safety profile of atabecestat during 6 months of treatment. This was followed by the longer term ALZ2004 extension study that consisted of 2 sequential treatment phases: a 12-month double-blind placebo-controlled treatment phase of atabecestat, followed by an open-label (OL) active treatment phase.

In the ALZ2004 extension study, the effects of atabecestat relative to placebo over time on cognition, performance of everyday functions, and brain volume as assessed by MRI were evaluated as secondary endpoints. In both studies, secondary and exploratory endpoints included the effect of treatment on the change in CSF levels of downstream biomarkers, including Aβ fragments (Aβ_1–37_, Aβ_1–38_, Aβ_1–40_, Aβ_1–42_), soluble APP (sAPP) fragments (sAPPα, sAPPβ, and total sAPP), total tau (t-tau), and phosphorylated tau (p-tau_181_).

## Methods

### Study population and selection criteria

The ALZ2002 parent study (NCT02260674) and the ALZ2004 extension study (NCT02406027) were multi-center, double-blind, placebo-controlled, randomized, multiple dose safety and tolerability studies in patients in the early (predementia) AD spectrum conducted in sites in Belgium, France, Germany, Netherlands, Sweden, and Spain from February 2016 to June 2018.

The parent ALZ2002 study population consisted of men and women who entered the study either directly as new patients or after completing 4 weeks of double-blind treatment in study ALZ1005, a phase 1 placebo-controlled study in participants meeting the same inclusion criteria [5]. The ALZ2002 patient population included asymptomatic individuals diagnosed as preclinical AD (aged 65 to 85 years) who were cognitively and functionally unimpaired with a Clinical Dementia Rating (CDR) global scale score of 0 and those diagnosed with MCI due to AD (aged 50 to 85 years) who had mild cognitive impairment but were functionally unimpaired with CDR global score of 0.5. Those with CDR global scores higher than 0.5 were excluded. Participants were considered otherwise healthy for their age with a body mass index (BMI) between 18 and 35 kg/m^2^.

Participants were excluded if they had any of the following: a diagnosis of dementia, or another degenerative brain disorder that can cause dementia; evidence of familial autosomal dominant AD, or any brain disease other than potential very early signs of AD; any other abnormality that could explain cognitive deficit such as vitamin B_12_ or folic acid deficiency, vascular encephalopathy, or strokes including lacunae; any finding on magnetic resonance imaging (MRI) of brain disease, other than mild hippocampal atrophy or typical age-related mild white matter hyperintensity; a Rosen modified Hachinski ischemic scale score of > 4; any contraindications for MRI; chromosome 21 trisomy (Down syndrome); major depression, as defined by the most current Diagnostic and Statistical Manual of Mental Disorders (DSM) criteria; or liver or renal insufficiency, or other clinically significant medical disorders. Treatment of stable medical conditions including the use of cognitive enhancers (e.g., cholinesterase inhibitors or memantine) was allowed, if started ≥ 30 days prior to the first study dose.

All participants had evidence of amyloid pathology at screening based on low CSF Aβ_1–42_ level having a concentration below the laboratory-specific cut-off value of 600 ng/L, analyzed in one central laboratory using INNOTEST® β-AMYLOID_1–42_ assay (Fujirebio, Ghent, Belgium) [6, 7] or by a positive amyloid positron emission tomography (PET) scan at screening based on visual inspection of PET imaging scan performed at a core imaging laboratory.

#### Study design

The ALZ2002 parent study included a 90-day screening period, followed by 6 months of double-blind treatment randomized 1:1:1 to placebo or atabecestat 10 mg or 50 mg once daily (given as 2 identical tablets of placebo, 5 mg, or 25 mg). Individuals that had completed the ALZ1005 study continued the same blinded treatment to which they had originally been randomized. After completion of month 6 of the ALZ2002 study, participants had the option to enroll in the ALZ2004 extension study; otherwise, they returned for a follow-up visit 7 to 28 days after last dose. Those who had progressed to dementia (CDR global score ≥ 1) in ALZ2002 were not eligible for enrollment in the ALZ2004; in case of progression to a dementia state during the course of ALZ2004, a renewed consent to remain in the study was obtained from the participant or a representative, provided they chose to continue and the investigator judged potential benefits of treatment outweighed foreseeable risks for that participant.

**Fig. 1:**
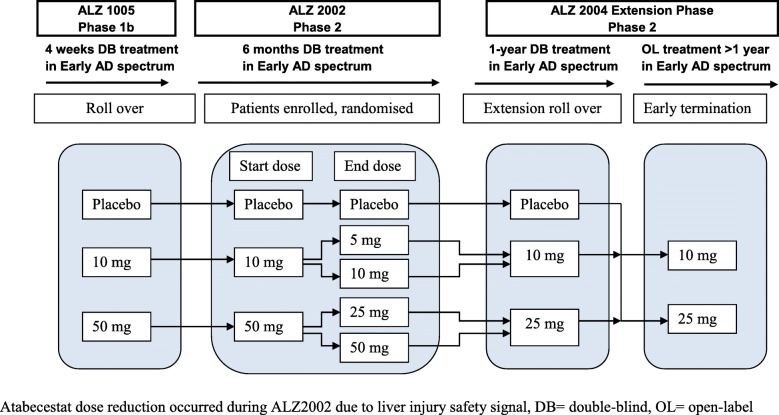
Trial design and treatment sequence of atabecestat and placebo. Atabecestat dose reduction occurred during ALZ2002 due to liver injury safety signal; DB double-blind, OL open-label

ALZ2004 consisted of 2 sequential treatment periods. In the initial 12-month double-blind phase, participants who were originally assigned to 50 mg atabecestat once daily in ALZ2002 were reduced to 25 mg, while those receiving 10 mg atabecestat or placebo continued their previously assigned treatment. This was followed by an open-label phase, where participants on 25 mg continued to receive that dosage, those on atabecestat 10 mg were decreased to 5 mg, and those on placebo were randomized with equal probability to 5 or 25 mg. The rationale for the 5-mg dosage was to identify a dose that may not require monitoring of hepatic enzymes, while still providing adequate target CSF Aβ_1–40_ reduction of 52% (95% CI 27–72%) [4]. The sequence of dosing in all phases of ALZ2002 and ALZ2004 is summarized graphically in Fig. 1.

Approximately 3 months after the completion of enrollment in study ALZ2002, the design of the study was substantially amended due to observations of elevated liver enzymes in some patients. Participants who had not yet completed ALZ2002 had a reduction in dosage of blinded study drug from 2 to 1 tablet per day (thus, from 50 mg, 10 mg, or placebo to 25 mg, 5 mg, or placebo, respectively). More frequent monitoring of serum chemistry and rules for stopping treatment were implemented for alanine aminotransferase (ALT) or aspartate aminotransferase (AST) > 8× upper limit of normal (ULN), ALT or AST > 5× ULN for more than 2 weeks, ALT or AST > 3× ULN and total bilirubin > 2× ULN or international normalized ratio > 1.5, or ALT or AST > 3× ULN with the appearance of fatigue, nausea, vomiting, right upper quadrant pain or tenderness, fever, rash, and/or eosinophilia (> 5%) [8]. Because of the continued high frequency of elevated liver enzymes observed in this study as well as in a separate parallel running phase 2b/3 trial of atabecestat in preclinical AD (the EARLY study, ALZ2003, NCT02569398), the sponsor judged that the risks did not merit continued treatment and dosing in all atabecestat trials was terminated in May 2018.

### Assessments

#### Cognition

Cognitive assessments performed prior to the initiation of treatment in ALZ2002 included the CDR-Sum of Boxes (CDR-SB), from which a global score was calculated to establish eligibility, the Mini-Mental State Examination (MMSE), the Repeatable Battery for the Assessment of Neuropsychological Status (RBANS) (performed twice, with the second session serving as the baseline measurement), and the California Verbal Learning Test—second edition [CVLT-II]) performed prior to dosing on day 1 (see Supplementary material for descriptions). The CDR-SB, MMSE, and RBANS were repeated at the 6-month visit in ALZ2002. Those participants entering ALZ2004 then had the CDR-SB and MMSE repeated at week 52 in the double-blind treatment period and thereafter every 48 weeks in the open-label period. The RBANS was repeated at weeks 24 and 52 in the double-blind period and thereafter every 24 weeks in the open-label period. The CVLT was repeated only at week 12 in the double-blind period. A patient-reported outcome, the Cognitive Function Index (CFI), was first administered on day 1 of ALZ2004 and was repeated at weeks 24 and 52 in the double-blind period and thereafter every 24 weeks in the open-label period.

#### Biomarkers

The pharmacodynamic effects of atabecestat on BACE1 activity were evaluated through effects relative to baseline on CSF concentrations of Aβ fragments (Aβ_1–37_, Aβ_1–38_, Aβ_1–40_, and Aβ_1–42_), with Aβ_1–40_ being the most abundant and primary determinant of atabecestat activity. A qualified Janssen multiplex immunoassay based on Meso Scale Discovery (MSD) (Gaithersburg, MD, USA) electrochemiluminescence (ECL) detection technology was utilized for simultaneous detection of all 4 Aβ fragments in CSF and plasma [9, 10]. In addition, exploratory measurements of CSF levels of sAPP fragments (sAPPα, sAPPβ) and other CSF biomarkers (i.e., CSF p-tau and t-tau) were performed by a central laboratory as previously described by Timmers et al. [4]. Potential atabecestat treatment effects on brain volume were assessed with MRI at baseline and month 6 in ALZ2002 and at double-blind period weeks 24 and 52 and thereafter every 48 weeks in ALZ2004, using the boundary shift integral method [11].

#### Pharmacokinetics

Sparse plasma pharmacokinetic (PK) samples were collected at pre-dose of baseline and days 28, 56, 84, 112, 140, and 168 (month 6). As some participants in ALZ2002 entered the study from ALZ1005, data from the 2 studies were pooled in order to perform a combined population PK analysis. PK samples from study ALZ2004 were not included in the analysis. The PK dataset contained a total of 702 PK samples from 90 individuals. Population PK modeling was performed on the PK dataset in order to derive individual estimates of exposure (area under the plasma concentration-time curve at steady state, AUC_0–24 h_, calculated as dose/(CL/F) where CL/F is the individual estimate of oral clearance) for the participants included in the PK dataset.

#### Safety assessments

All participants who received study treatment were included in the safety analysis population. Safety and tolerability were assessed by recording adverse events (AEs) and clinically significant abnormalities on clinical laboratory tests (hematology, biochemistry, including liver function tests), 12-lead electrocardiogram, vital signs, and abnormalities on physical or neurological exams, and potential dermatologic and ophthalmologic abnormalities (e.g., melanin deposition changes and retinal abnormalities). In order to monitor for potential changes in melanin deposition and retinal abnormalities [12], all included participants had a baseline ophthalmological examination, optical coherence tomography, and examination of the skin by a dermatologist. These examinations were repeated in case of abnormality at baseline or new visual signs or symptoms.

MRI was used to monitor for amyloid-related imaging abnormalities (ARIA) with edema or effusion (ARIA-E) or with hemosiderin (ARIA-H), as previously reported with several anti-amyloid monoclonal antibodies [13]. AEs were coded using Medical Dictionary for Regulatory Activities (MedDRA) version 19.1 or later. Possible effects on anxiety or mood were assessed by the Geriatric Depression Scale, the State-Trait Anxiety Inventory, and the Columbia Suicide Severity Rating Scale (C-SSRS), performed at screening and on scheduled visits thereafter.

#### Statistical analysis

No formal hypothesis testing was planned, and sample sizes for the studies were not based on formal statistical testing. However, using assumptions that an AE occurred at the rates of 1%, 5%, or 10% in a single treatment group, then the chances of observing such an AE among 30 participants receiving that treatment was estimated to be 26%, 79%, or 96%, respectively. Thus, planned enrollment of 100 participants (approximately 33 per group) was considered sufficient for clinical judgment of safety and tolerability of atabecestat.

Percent change from baseline in CSF concentration of each of the Aβ fragments (Aβ_1–37_, Aβ_1–38_, Aβ_1–40_, and Aβ_1–42_), of sAPPα and sAPPβ, and of p-tau and t-tau was summarized by box and whisker plots separately by treatment group and by baseline CDR status (i.e., preclinical AD, MCI due to AD) for month 6 in ALZ2002 and week 52 in the double-blind period of ALZ2004.

Change in cognition at week 52 of the double-blind period in ALZ2004 relative to baseline in ALZ2002 was analyzed with an ANCOVA model which included the treatment group, actual baseline CDR status, and baseline score as a covariate. For non-biomarker assessments, baseline for the ALZ2004 double-blind period safety analysis set was the value taken prior to the first study drug dose in the ALZ2002. For the open-label period in ALZ2004, baseline was the last value taken before the first dose in that period.

## Results

### Demographics, baseline characteristics, and disposition

A total of 114 participants were enrolled in the ALZ2002 parent study, including 27 who entered from ALZ1005 (placebo, *n* = 11; atabecestat 10 mg, *n* = 8; and atabecestat 50 mg, *n* = 8). Ninety participants were classified with MCI due to AD and 24 with preclinical AD. Participant demographics, apolipoprotein E ε4 (*APOE* ε4) carrier status, and global CDR score are summarized in Table 1 by treatment group at the start of the study. These baseline characteristics were generally well balanced across treatment groups. Nearly all participants were Caucasians. *APOE* genotype was available for 69% of individuals: majorities were carriers of the ε4 allele. Baseline/day 1 pre-dose scores for clinical scales and cognitive assessments by treatment groups at start and by the CDR diagnostic group are shown in Table 2. In general, scores were comparable across treatment groups; however, patients classified with MCI due to AD showed more pronounced impairment on the MMSE, CDR-SB, RBANS, and CVLT-II, compared to those with preclinical AD.

**Table 1:** Baseline demographic characteristics of patients enrolled in the ALZ2002 (safety set)

Baseline characteristics	Treatment group at start ALZ2002			
	Placebo	10 mg	50 mg	Total
***N***	39	37	38	114
**Sex, men,*n*(%)**	16 (41.0)	18 (48.6)	20 (52.6)	54 (47.4)
**Age, years**				
**Mean (SD)**	70.3 (5.38)	70.9 (6.58)	68.1 (8.55)	69.8 (6.99)
**Range**	59; 80	52; 81	50; 82	50; 82
**Race**				
**White,*n*(%)**	37 (94.9)	36 (97.3%)	38 (100.0)	111 (97.4)
**Other,*n*(%)**	2 (5.1)	1 (2.7)	0 (0.0)	3 (2.6)
***APOE*ε4 carrier status known,*n***	27	30	22	79
**Yes,*n*(%)**	17 (63.0)	18 (60.0)	10 (45.5)	45 (57.0)
**No,*n*(%)**	10 (37.0)	12 (40.0)	11 (50.0)	33 (41.8)
**Preclinical AD, CDR = 0,*n*(%)**	7 (17.9)	8 (21.6)	9 (23.7)	24 (21.1)
**MCI due to AD, CDR = 0.5,*n*(%)**	32 (82.1)	29 (78.4)	29 (76.3)	90 (78.9)

**Table 2:** Baseline clinical and cognitive characteristics of patients in parent study ALZ2002 by treatment group at start and by their Clinical Dementia Rating classification (safety analysis set)

Baseline characteristics	Treatment group at start		
Clinical and Cognitive Scales	Placebo (***N*** = 39)	Atabecestat	
		10 mg (***N*** = 37)	50 mg (***N*** = 38)
**CDR-SB**^†^**,*N***	39	37	38
**All mean (SD)**	1.5 (1.25)	1.6 (1.24)	1.6 (1.09)
**Preclinical AD,*N***	7	8	9
**Mean (SD)**	0.1 (0.19)	0.4 (0.73)	0.8 (0.97)
**MCI due to AD,*N***	32	29	29
**Mean (SD)**	1.8 (1.16)	1.9 (1.16)	1.8 (1.04)
**CVLT-II, long-delay recall**‖**,*N***	36	36	37
**All mean (SD)**	11.7 (9.4)	10.3 (9.04)	12.4 (8.48)
**Preclinical AD,*N***	7	8	9
**Mean (SD)**	19.7 (7.67)	15.8 (12.29)	20.7 (8.14)
**MCI due to AD,*N***	29	28	28
**Mean (SD)**	9.7 (8.46)	8.8 (7.45)	9.8 (6.80)
**MMSE subscale scores**^‡^**,*N***	39	37	38
**All mean (SD)**	26.3 (2.90)	24.9 (4.14)	26.2 (2.47)
**Preclinical AD,*N***	7	8	9
**Mean (SD)**	29.0 (1.15)	27.8 (2.55)	27.8 (2.64)
**MCI due to AD,*N***	32	29	29
**Mean (SD)**	25.8 (2.84)	24.1 (4.18)	25.7 (2.24)
**RBANS—total scale score**^**¶**^**,*N***	39	37	38
**All, mean (SD)**	80.2 (17.10)	72.4 (21.99)	80.1 (17.44)
**Preclinical AD,*N***	7	8	9
**Mean (SD)**	99.7 (14.68)	91.4 (23.89)	92.9 (17.49)
**MCI due to AD,*N***	32	29	29
**Mean (SD)**	75.9 (14.55)	67.2 (18.66)	76.2 (15.68)

^†^The Clinical Dementia Rating-Sum of Boxes with scores ranging from 0 to 18 with higher scores indicating more pronounced impairment

^‖^The California Verbal Learning Test—second edition long-delay recall subtest is derived as the sum of free recall and cued recall 20 min after the initial presentation of a word list. The lower the score, the more pronounced the impairment

^‡^The Mini-Mental State Examination is a questionnaire that rates participants on orientation (total score, 10), registration (total score, 3), attention, calculation (total score, 5), recall (total score, 3), and language (total score, 9). The maximum score is 30. The lower the score, the more pronounced the impairment

^¶^The Repeatable Battery for the Assessment of Neuropsychological Status subsets included attention index, language index, visuospatial/constructional index, immediate memory index, and delayed memory index. The lower the score, the more pronounced the impairment

**Fig. 2:**
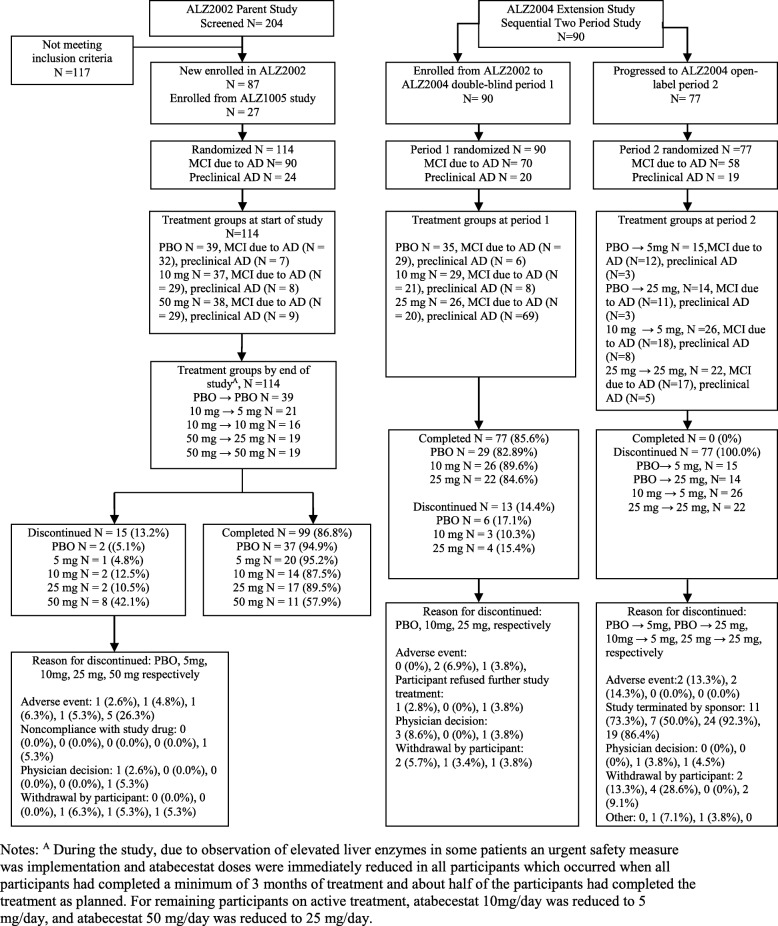
Participant dispositions for ALZ2002 parent and ALZ2004 extension studies. ^A^During the study, due to the observation of elevated liver enzymes in some patients, an urgent safety measure was implemented and atabecestat doses were immediately reduced in all participants which occurred when all participants had completed a minimum of 3 months of treatment and about half of the participants had completed the treatment as planned. For the remaining participants on active treatment, atabecestat 10 mg/day was reduced to 5 mg/day, and atabecestat 50 mg/day was reduced to 25 mg/day

By the time the dosage of blinded study drug was reduced due to elevated hepatic enzymes, all participants had been enrolled in ALZ2002 and had completed at least 3 months of treatment. The resulting treatment exposures over 6 months (mean daily dose) were relatively similar between those that underwent dosage reduction and those that completed month 6 on the originally assigned dosage (9 mg vs 10 mg for 5 mg [dose-reduced] and 10 mg [completed on original dose], and 40 mg vs 50 mg for 25 mg [dose-reduced] and 50 mg [completed on original dose], respectively). Participant disposition and study completion for the ALZ2002 study are shown in Fig. 2. Of the 114 participants enrolled, 99 (87%) completed 6 months of double-blind treatment in ALZ2002. The most common reason for discontinuation of the study drug was an AE in 9 (7.9%) participants.

In ALZ2004, 90 participants enrolled into the double-blind period of the study; 77 (85.6%) of these completed the double-blind period and progressed to the open-label period (Fig. 2). Of the 29 participants who received placebo in the double-blind period and progressed to open-label, 15 were re-randomized to atabecestat 5 mg and 14 to atabecestat 25 mg. Twenty-six participants who had received 10 mg in the double-blind period were reduced to 5 mg in open-label, while 22 who had received 25 mg remained on that dosage. At the time the decision was made by the sponsor to discontinue all dosing (17 May 2018), 62 of 77 (80.5%) participants were still receiving atabecestat in the open-label period, while the remainder had already discontinued.

### Fluid and imaging (vMRI) biomarker analysis

**Fig. 3:**
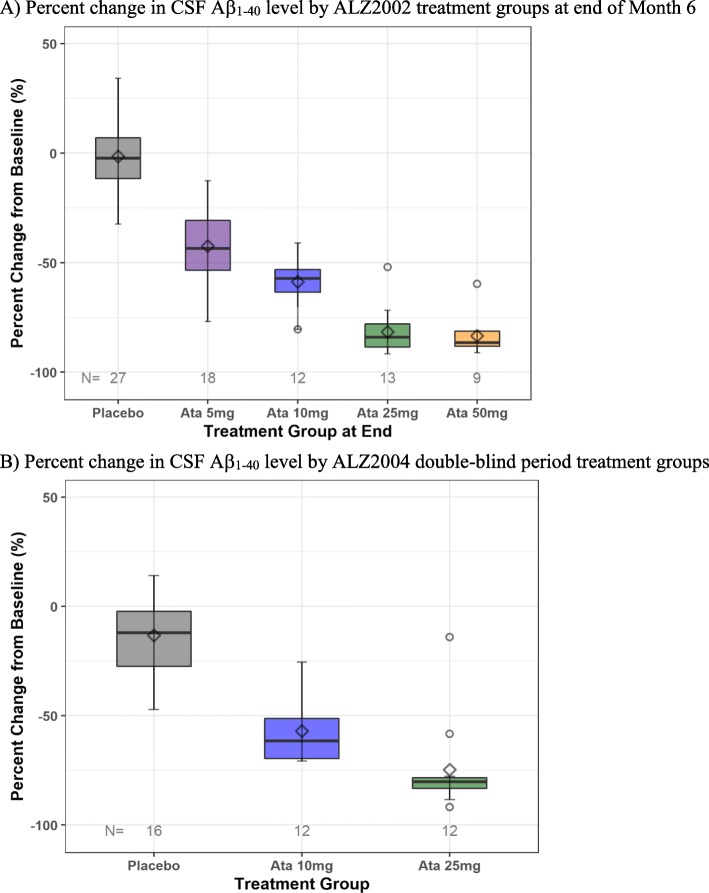
**a** Box-whisker plots of percent change from baseline for CSF Aβ_1–40_ biomarker level by final dose groups at the end of month 6 of atabecestat treatment in ALZ2002 early AD population. **b** Percent change from baseline time profile for CSF Aβ_1–40_ levels to 52 weeks in ALZ2004 double-blind period. The line inside the box represents the median value, and the symbol represents the mean value. The outer box borders represent the lower and upper quartile (25th and 75th percentiles of the data)

In the ALZ2002 study, at month 6 (day 168), there was a dose-dependent mean (standard deviation [SD]) percent reduction from baseline in the CSF Aβ_1–40_ levels: − 42.4% [15.3] for 5 mg (dose-reduced), − 58.7% [10.5] for 10 mg (original dose), − 81.6% [10.8] for 25 mg (dose-reduced), and − 83.3% [9.5] for 50 mg (original dose) groups as shown in Fig. 3a. Similar and proportionate effects of dosage on reductions from baseline were observed for CSF Aβ_1–37_, Aβ1_− 38_, and Aβ_1–42_, fragments (data not shown). Plasma Aβ_1–40_ levels were reduced during the treatment period with atabecestat compared with their baseline similar to CSF Aβ_1–40_ (Supplementary Figure 1A). No change was observed in the placebo group. There were no significant changes on CSF Aβ fragment levels on placebo. There were no apparent differences in the change from baseline for CSF Aβ_1–40_ and Aβ_1–42_ between *APOE* ε4 carriers or non-carriers.

**Fig. 4:**
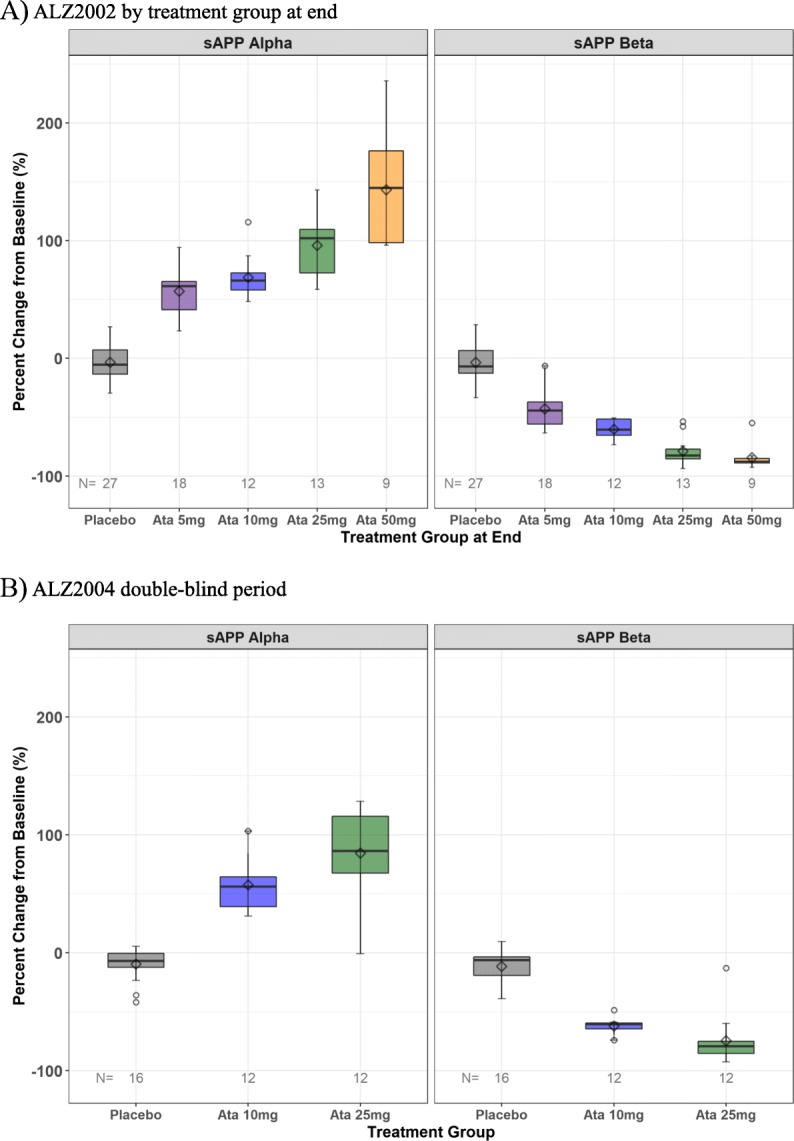
**a** Box-whisker plots of percent change from baseline for CSF sAPPα and sAPPβ biomarkers by final dose groups at month 6 of atabecestat treatment in ALZ2002 and **b** for percent change from ALZ2002 baseline for CSF sAPPα and sAPPβ to week 52 in the ALZ2004 double-blind period

At month 6, there was a dose-dependent decrease in the CSF sAPPβ and, in contrast, a dose-dependent increase in sAPPα fragment levels as compared to their baseline levels, which is consistent with atabecestat mode of action in inhibition of β-secretase proteolytic cleavage of APP (Fig. 4a). No change in sAPPα and sAPPβ was observed in patients treated with placebo. There was no change in CSF levels of t-tau and p-tau_181_ over the 6-month treatment period across the atabecestat and placebo groups.

Box and whisker plots of change from baseline value in ALZ2002 to week 52 in ALZ2004 double-blind period are shown in Fig. 3b for CSF Aβ_1–40_, in Supplementary Figure 1B for plasma Aβ_1–40_, and in Fig. 4b for sAPPα and sAPPβ. The magnitude of change from baseline increased with the dose administered and was similar to that of month 6 in ALZ2002. No relevant changes were observed for tau proteins.

Summary statistics of changes in the whole brain, total hippocampal, and ventricular volume during the 52-week double-blind period in ALZ2004, quantified by BSI, are presented in Table 3. Overall, as shown, numerical decreases in the whole brain and hippocampal volumes and increases in ventricular volumes from baseline were greater in participants with MCI due to AD relative to preclinical AD, though there were no clear differences related to treatment.

**Table 3:** Changes in brain volumes from baseline in ALZ2002 to the end of the double-blind period in ALZ2004, by treatment group (safety analysis set)

Dosage	Region	Preclinical AD				MCI due to AD			
		Double blind week 24		Double blind week 52		Double blind week 24		Double blind week 52	
		***n***	Mean (SD)	***n***	Mean (SD)	***n***	Mean (SD)	***n***	Mean (SD)
**Placebo**	Total hippocampus	6	− 61.183 (152.8617)	4	− 192.691 (286.0535)	23	− 148.955 (110.0055)	16	− 208.173 (139.2326)
	Bilateral ventricles	6	1213.479 (1297.2666)	4	2384.305 (1765.8039)	23	1877.688 (1868.2961)	16	2554.255 (1619.2302)
	Whole brain	6	− 4513.446 (6744.6900)	4	− 3966.134 (6897.1749)	21	− 5202.949 (5310.6112)	15	− 6398.921 (6057.7712)
**10 mg**	Total hippocampus	8	− 130.472 (133.3899)	4	− 314.289 (190.6022)	18	−130.601 (100.9252)	16	− 308.766 (198.3581)
	Bilateral ventricles	8	1317.472 (1089.6703)	4	3254.365 (1326.9982)	18	1294.812 (1190.6962)	16	3349.908 (2563.2821)
	Whole brain	8	− 7197.703 (7409.7046)	4	− 17,698.910 (5463.6862)	17	− 5736.212 (6275.3521)	16	− 10,815.252 (9814.0704)
**25 mg**	Total hippocampus	5	−12.221 (92.4995)	5	− 129.273 (81.0670)	16	− 122.374 (86.0882)	13	− 235.946 (145.2177)
	Bilateral ventricles	5	538.445 (965.7828)	5	1999.089 (1514.6993)	16	1399.116 (1085.9684)	13	2853.589 (1945.9111)
	Whole brain	5	− 1144.502 (7511.2280)	5	− 4524.925 (6112.8815)	14	− 6634.464 (5868.5297)	13	− 9624.766 (8707.2218)

All values are changes in regional volume measured by the boundary shift integral (BSI) method, relative to baseline in ALZ2002; units are cubic millimeters

### Pharmacokinetics

The plasma PK data were described by a two-compartment model, with distribution of drug between a central (i.e., plasma) compartment and a peripheral compartment, and with linear elimination from the central compartment. Absorption was described as a linear process characterized by sequential zero- and first-order absorption from the depot compartment into the central compartment. This linear model adequately described the data and confirmed the dose-proportional PK of atabecestat in the dose range between 5 and 50 mg [4, 5]. Individual estimates of AUC_0–24 h_ by dose are summarized in Supplementary Table S1. Participants who underwent dose reduction were included in the summary statistics with both their starting and final doses. Overall, the exposure parameters were in line with previous studies at corresponding doses [4, 5].

### Clinical scales and cognitive effects

**Fig. 5:**
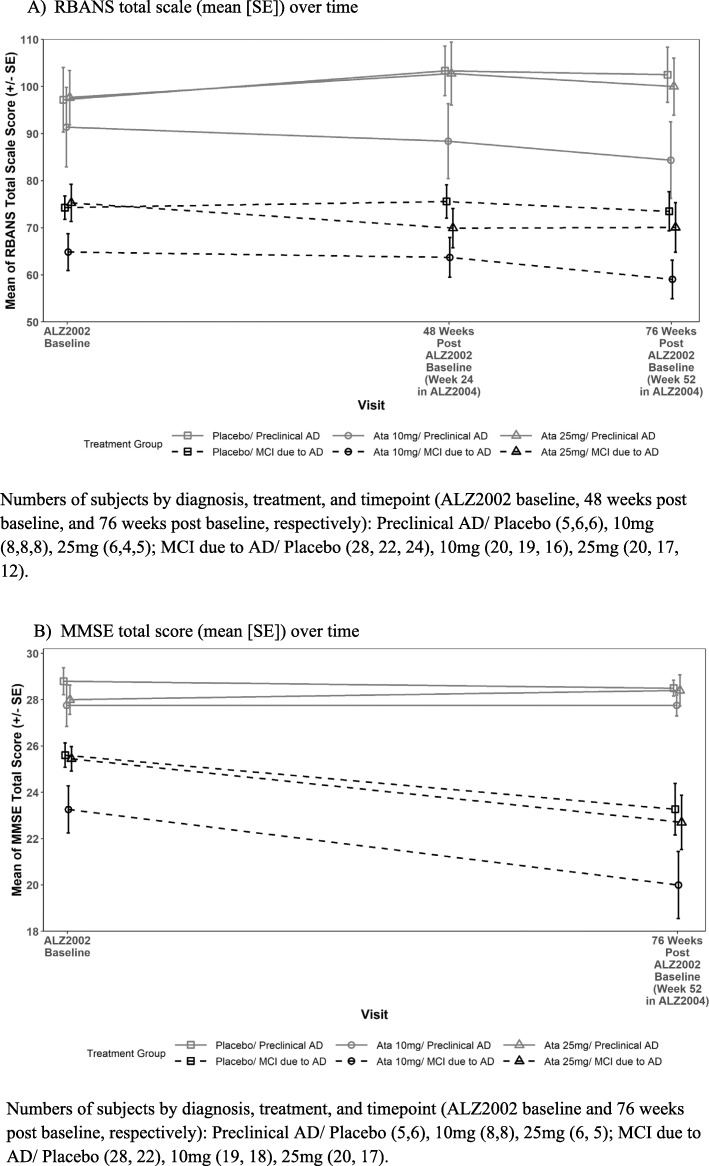
**a** RBANS total scale and **b** MMSE total score at baseline in ALZ2002 and at the end of the double-blind period in ALZ2004, by baseline CDR status and by treatment group

Mean MMSE and RBANS total scores from baseline in ALZ2002 to the end of the double-blind period in ALZ2004 are presented in Fig. 5a and b, respectively, by the treatment and diagnostic groups. ANCOVA adjusting for baseline score and diagnosis revealed that the differences in LS means relative to placebo were minimal for the MMSE (10 mg, 0.43 [90% CI − 1.25; 2.11], *p* = 0.6699; 25 mg, 0.55 [90% CI − 1.09; 2.20], *p* = 0.5751) but were numerically worse with atabecestat compared to placebo for total RBANS (10 mg, − 4.05 [90% CI − 8.68; 0.59], *p* = 0.1499; 25 mg, − 5.60 [90% CI − 10.47; − 0.72], *p* = 0.0600) (Supplementary Table S2).

Mean (SD) values and standard error (SE) for change from baseline for CDR-SB, and CFI-participant and partner by the treatment group are summarized in Supplementary Table S3. There were no consistent differences related to the treatment effect. Scores on the CDR-SB worsened over time during the treatment period. No consistent change was seen for the CVLT though the interval between tests was shorter (9 months). Of note, CFI-partner scores did not change for preclinical AD but increased (worsened) for MCI due to AD, while CFI-participant scores showed slight improvement regardless of the baseline diagnosis, consistent with the observation that patients with MCI lose insight into their cognitive worsening, compared to the observation made by independent raters [14].

### Clinical safety

#### Adverse events other than abnormal liver enzymes

Safety analyses included data from all randomized participants who received at least 1 dose of study drug. A summary of the incidence of treatment-emergent (TE) adverse events (AEs) by treatment groups for ALZ2002 and ALZ2004 is shown in Table 4. Overall, 81/114 (71.1%) of the participants experienced at least 1 TEAE in ALZ2002. The numbers of participants with at least 1 AE, serious AE, or AE leading to discontinuation were more commonly seen on the higher atabecestat dosages in ALZ2002 and the double-blind period in ALZ2004 (50 mg or 25 mg, respectively). Only 1 participant receiving placebo discontinued because of an AE during ALZ2004 double-blind treatment, but only participants transitioning from placebo to atabecestat discontinued because of AEs in the open-label period. There was 1 death in the study: a 77-year-old woman in the ALZ2002 atabecestat 10-mg group died on day 170 of a cholangiocarcinoma which the investigator expected to have been pre-existent, not related to study drug, and not likely to have developed within the short treatment duration.

**Table 4:** Summary of the overall treatment-mergent adverse events by the treatment group for parent study ALZ2002 and extension study ALZ2004 (safety analysis set)

Safety analysis set	Treatment group at start ALZ2002				Treatment group in ALZ2004								
	Double-blind parent				Double-blind period^†^				Open-label period^‡^				
	Placebo	10 mg	50 mg	Total	Placebo	10 mg	25 mg	Total	Atabecestat				
									PBO→5 mg	PBO→25 mg	10 mg → 5 mg	25 mg	Total
***N***	39	37	38	114	35	29	26	90	15	14	26	22	77
**Total patients with TEAEs,*n*(%)**	27 (69.2)	23 (62.6)	31 (81.6)	81 (71.1)	22 (62.9)	15 (51.7)	21 (80.8)	58 (64.4)	10 (66.7)	11 (78.6)	19 (73.1)	16 (72.7)	56 (72.7)
**Serious TEAEs,*n*(%)**	4 (10.3)	2 (5.4)	8 (21.1)	14 (12.3)	3 (8.6)	4 (13.8)	4 (15.4)	11 (12.2)	2 (13.3)	4 (28.6)	0 (0)	1 (4.5)	7 (9.1)
**TEAEs leading to death,*n*(%)**	0 (0)	1 (2.7)	0 (0)	1 (0.9)	0 (0)	0 (0)	0 (0)	0 (0)	0 (0)	0 (0)	0 (0)	0 (0)	0 (0)
**Any AE leading to discontinuation or withdrawal (%)**	1 (2.6)	2 (5.4)	5 (13.2)	8 (7.0)	0 (0)	2 (6.9)	1 (3.8)	3 (3.3)	2 (13.3)	2 (14.3)	0 (0)	0 (0)	4 (5.2)

*TEAE* treatment-emergent adverse events, coded using MedDRA version 21.0

^†^Adverse events (AEs) with onset on or after the first dose of study drug in ALZ2004 through the last dose in period 1 are included. For a participant who withdrew during period 1, adverse events through the day of the last dose plus 7 days are included

^‡^AEs with onset on or after the first dose of study drug in period 2 of ALZ2004 through the day of the last dose in period 2 plus 7 days are included

The most common AEs (occurring in at least 2 participants and ≥ 5% in any one treatment group) are presented for each study period in Table 5. In ALZ2002, diarrhea was the most frequently reported TEAE associated with active treatment, occurring in 10 individuals (8.1% of those for 10 mg and 18.4% of those for 50 mg). Other frequent AEs, occurring more commonly for atabecestat than placebo, included gastroesophageal reflux and influenza, 2 (5.3%) participants each for 50 mg. AEs that occurred more commonly on placebo included nasopharyngitis (12.8%), urinary tract infection (10.3%), and headache (10.3%), as well as vomiting, hypertension, and syncope, occurring in 7.7% each. In the double-blind period of ALZ2004, AEs occurring more commonly on atabecestat included bronchitis (11.5%) and nasopharyngitis (7.7%) for the 25-mg dosage, and falls, depressive symptoms, and malaise (6.9% each) for the 10-mg dosage. Cataracts (17.1%) occurred more commonly on placebo.

**Table 5:** Incidence of the most frequent (> 5%) adverse events occurring in the ALZ2002 and the ALZ2004 double-blind and open-label periods

Body system/preferred term	Treatment group				
**ALZ2002 (double-blind)**	**Placebo**		**Ata 10 mg**	**Ata 50 mg**	**Total**
***N***	39		37	38	114
**Diarrhea**	2 (5.1%)		3 (8.1%)	7 (18.4%)	12 (10.5%)
**Nasopharyngitis**	5 (12.8%)		3 (8.1%)	3 (7.9%)	11 (9.6%)
**Headache**	4 (10.3%)		2 (5.4%)	2 (5.3%)	8 (7.0%)
**Back pain**	2 (5.1%)		2 (5.4%)	2 (5.3%)	6 (5.3%)
**Hypertension**	3 (7.7%)		2 (5.4%)	1 (2.6%)	6 (5.3%)
**Transaminases increased**	0		2 (5.4%)	3 (7.9%)	5 (4.4%)
**Urinary tract infection**	4 (10.3%)		1 (2.7%)	0	5 (4.4%)
**Influenza**	1 (2.6%)		0	2 (5.3%)	3 (2.6%)
**Syncope**	3 (7.7%)		0	0	3 (2.6%)
**Vomiting**	3 (7.7%)		0	0	2 (2.6%)
**Cataract**	2 (5.1%)		0	0	2 (1.8%)
**Gastroesophageal reflux disease**	0		0	2 (5.3%)	2 (1.8%)
**Alanine aminotransferase increased**	0		2 (5.4%)	3 (7.9%)	5 (4.4%)
**Vitamin B**_**12**_**decreased**	2 (5.1%)		0	0	2 (1.8%)
**ALZ2004, double-blind period**	**Placebo**		**Ata 10 mg**	**Ata 25 mg**	**Total**
***N***	35		29	26	90
**Cataract**	6 (17.1%)		0	2 (7.7%)	8 (8.9%)
**Nasopharyngitis**	2 (5.7%)		2 (6.9%)	2 (7.7%)	6 (6.7%)
**Bronchitis**	1 (2.9%)		1 (3.4%)	3 (11.5%)	5 (5.6%)
**Fall**	2 (5.7%)		2 (6.9%)	0	4 (4.4%)
**Diarrhea**	1 (2.9%)		1 (3.4%)	1 (3.8%)	3 (3.3%)
**Depressive symptom**	0		2 (6.9%)	0	2 (2.2%)
**Insomnia**	0		0	2 (7.7%)	2 (2.2)
**Malaise**	0		2 (6.9%)	0	2 (2.2%)
**ALZ2004 open-label period**	**Placebo/Ata 5 mg**	**Placebo/Ata 25 mg**	**Ata 5 mg**	**Ata 25 mg**	**Total**
***N***	15	14	28	22	77
**Nasopharyngitis**	1 (6.7%)	3 (21.4%)	3 (11.5%)	1 (4.5%)	8 (10.4%)
**Headache**	0	2 (14.3%)	3 (11.5%)	1 (4.5%)	6 (7.8%)
**Back pain**	1 (6.7%)	0	0	3 (13.6%)	4 (5.2%)
**Pneumonia**	0	2 (14.3%)	2 (7.7%)	0	4 (5.2%)
**Cataract**	0	0	2 (7.7%)	1 (4.5%)	3 (3.9%)
**Macular fibrosis**	0	0	0	2 (9.1%)	2 (2.6%)
**Depression**	2 (13.3%)	0	0	1 (4.5%)	3 (3.9%)
**Nightmare**	2 (13.3%)	1 (7.1%)	0	0	3 (3.9%)
**Insomnia**	0	0	0	2 (9.1%)	2 (2.6%)
**Confusional state**	0	1 (7.1%)	0	0	1 (1.3%)
**Delirium**	1 (6.7%)	0	0	0	1 (1.3%)
**Psychotic disorder**	0	1 (7.1%)	0	0	1 (1.3%)

Retinal AEs occurred in 2 participants receiving open-label 25 mg (macular fibrosis) and in 3 receiving placebo (retinal degeneration, retinal detachment, and retinal exudates, respectively). There were no changes on examination by the ophthalmologist that were considered possibly related to study drug. Pigmentary changes were seen in 1 individual in the 10-mg group in ALZ2002 (skin hypopigmentation) and in 2 in the placebo group in ALZ2004 (change in hair coloration).

Eight individuals endorsed statements related to suicidal ideation on the Columbia Suicide Severity Rating Scale (though none had plan or intent) which occurred in 1 individual on placebo, 1 on placebo and later 5 mg, 3 on 5 or 10 mg, and 2 on 25 mg. Sleep disorders have been reported for other BACE1 inhibitors in other trials [15]. Sleep-related complaints in this study included insomnia (1 patient on placebo, 1 on 10 mg, and 2 on 25 mg), nightmares (1 on placebo, 1 on 25 mg, and transitioning from placebo to 5 mg [2] or 25 mg [1]), parasomnias (1 on 10 mg), and rapid eye movement sleep abnormalities (1 on 25 mg).

#### AEs related to elevated liver enzymes

Hepatic-related AEs were reported only in the atabecestat treatment groups and not in the placebo group in ALZ2002 and in the double-blind period in ALZ2004. The reported AE terms included increased alanine aminotransferase (ALT), increased aspartate aminotransferase (AST), increased transaminases, increased hepatic enzymes, and increased gamma-glutamyl transferase (GGT). Five individuals, including 4 for 50 mg and 1 for 10 mg, had these reported as serious AEs during double-blind treatment in ALZ2002 and in the double-blind period in ALZ2004, and 2 had these reported as serious AEs transitioning from placebo to 5 mg or to 25 mg in open-label period. These individuals all discontinued treatment because of these AEs.

Treatment-emergent increases in ALT or AST (> 1 to < 2× ULN, 2 to < 3× ULN, and > 3X ULN) are presented in Table 6 for starting dosage in ALZ2002 and for the double-blind and open-label periods in ALZ2004. Mild increases (< 3× ULN) were seen more commonly on atabecestat than on placebo. Only 1 participant had an increase in ALT > 3× ULN while receiving placebo, due to acute cholecystitis. A total of 12 participants had an increase in ALT > 3× ULN while receiving atabecestat, including 5 in ALZ2002, 3 in the double-blind period of ALZ2004, and 4 in participants transitioning from placebo to 5 mg (1 case) or to 25 mg (3 cases).

**Table 6:** Incidence of treatment-emergent abnormal value of ALT or AST

**Study**	**ALZ2002**			**ALZ2004**						
**Period**	**Double-blind**			**Double-blind**			**Open-label**			
**Treatment group**	**Placebo**	**Ata 10 mg**	**Ata 50 mg**	**Placebo**	**Ata 10 mg**	**Ata 25 mg**	**Placebo/5 mg**	**Placebo/25 mg**	**5 mg**	**25 mg**
**AST or ALT,*n***	39	34	37	35	29	26	15	14	26	22
**> ULN to ≤ 2× ULN,*n*(%)**	0	5 (14.7)	8 (21.6)	2 (5.7)	1 (3.4)	7 (26.9)	4 (26.7)	2 (14.3)	3 (11.5)	7 (31.8)
**> 2× ULN to ≤ 3× ULN,*n*(%)**	0	2 (5.9)	1 (2.7)	0	2 (6.9)	1 (3.8)	0	2 (14.3)	0	0
**> 3× ULN,*n*(%)**	1 (2.6)	2 (5.9)	2 (5.4)	0	2 (6.9)	1 (3.8)	1 (6.7)	3 (21.4)	0	0

*ALT* alanine aminotransferase, *AST* aspartate aminotransferase, *ULN* upper limit of normal

For double-blind period 1, baseline is defined as the pre-dose baseline value from the preceding parent ALZ2002 study. For open-label period 2, baseline is defined as the last value taken on or before the day of the first dose of study drug in the open-label period

All cases of ALT or AST > 3× ULN occurred within the first year of exposure, including 7 between days 33 and 168, 4 between days 259 and 343, and 1 at day 201, 32 days after the last dose. In 3 cases, transaminases normalized with continued treatment; in 8, it resolved with discontinuation, and in 1 with abnormal ALT at baseline (2.7× ULN), it remained mildly elevated after discontinuation. All individuals were asymptomatic, except a 78-year-old man receiving 50 mg who presented on day 33 with a “flu-like” illness and a cholestatic pattern of abnormalities (ALT 5.4× ULN, alkaline phosphatase 3.6 ULN, and GGT 14.1× ULN). This resolved by day 56 following discontinuation of atabecestat, but recurred on day 89, in the setting of fever and other constitutional symptoms, a markedly elevated erythrocyte sedimentation rate, and temporal arteritis on biopsy. This resolved on a course of corticosteroids. A cholestatic pattern of liver enzymes is known to occur in approximately one-third of cases of temporal arteritis [16]. In all other cases, the pattern was hepatocellular, with ALT > AST. The highest elevations of ALT occurred in 1 participant receiving 50 mg (15.7× ULN) and in 1 receiving 25 mg (13.9× ULN). No abnormalities meeting “Hy’s law” criteria (ALT or AST > 3× ULN and total bilirubin > 2× ULN) were observed.

## Discussion

The effect of atabecestat on lowering CSF Aβ levels was dose-proportional and was maintained over a longer term in the extension study, confirming its pharmacodynamic effects of reducing the production of Aβ by central inhibition of BACE1 cleavage of APP. The Aβ reduction was consistent with previous findings from the ALZ1005 study which showed mean percent reduction from baseline in CSF Aβ_1–40_ of 67.3% and 89.9% for 10 mg and 50 mg, respectively, after 4 weeks of treatment [5]. This finding was not associated with statistically significant change in cognition or clinical scales, though a trend for a worsening of RBANS total score in the 10-mg and 25-mg groups relative to placebo was observed. There were no consistent changes in volumetric MRI related to treatment.

In this study, treatment-emergent increases in serum ALT, surpassing 3× ULN, were seen in 12 of 104 (11.5%) individuals exposed to atabecestat (including 8 of 75 in double-blind treatment and 4 of 29 participants transitioning from placebo to atabecestat). Though the frequency was similar for the higher and lower doses of atabecestat, there was a limited experience in those receiving 5 mg as the only dosage (only 14 participants). These AEs were nearly all asymptomatic and resolved either spontaneously or after discontinuation of treatment.

In a separate phase 2b/3 double-blind placebo-controlled study of atabecestat in preclinical AD (EARLY trial, ALZ2003), conducted concurrently with ALZ2004, mean decreases from baseline in PACC and RBANS scores (worsening) for participants on atabecestat treatment were statistically significant for the 25-mg group versus placebo at 6 and 12 months and 3 months, respectively [17]. The clinical significance of cognitive decline in atabecestat treatment groups relative to placebo is unclear, but similar results favoring placebo were reported with other BACE1 inhibitors such as verubecestat in phase 3 trials for prodromal AD and mild-to-moderate AD [15, 18]. Knopman [19] noted that these observations may suggest that lowering BACE1 activity could result in adverse effects on normal synaptic functions possibly due to excessive BACE1 inhibition and only a low degree of inhibition may be needed to decrease Aβ production. The cognitive changes described for several BACE inhibitors may be due to effects on other BACE substrates given diverse physiological function of BACE1 besides APP processing. While the relevance of BACE inhibitor’s nonselective blockage of BACE2, a close homolog of BACE1, remains unknown, it may theoretically lead to some AEs [20, 21]. Hence, the amount of Aβ reduction may not correlate with the effect on cognitive worsening and as such may warrant further investigation.

The EARLY trial had an active data safety monitoring board ensuring participant safety in the clinical trials. Because of the hepatic enzyme elevations observed in ALZ2002 as well as in EARLY, rigorous hepatic safety case management and hepatic safety algorithms had been implemented. With accumulation in the number and severity of hepatic enzyme elevations, the sponsor decided on 18 May 2018 to stop treatment in all atabecestat clinical trials.

Late phase clinical trials of other BACE1 inhibitors were recently terminated early or stopped for futility due to the lack of efficacy or for safety reasons, including studies of verubecestat (Merck, NJ, USA) in patients with mild-to-moderate AD (EPOCH study, NCT01739348) [15] and with amnestic MCI (APECS study, NCT 01953601) [18], and studies of lanabecestat (AstraZeneca, UK, and Eli Lilly IN, USA) in patients with MCI due to AD or mild AD dementia (AMARANTH NCT 02245737, DAYBREAK NCT02783573), [22]. Two phase 3 trials of elenbecestat (Biogen, MA, USA, and Eisai) in early AD spectrum (MISSION AD1 NCT02956486, MISSION AD2 NCT03036280) were expected to report findings by 2021 but were recently terminated for safety concerns. Although evidence of liver toxicity in patients does not appear to be a BACE inhibitor class effect, there were previous reports that phase 2 trials of LY-2886721 (Eli Lilly, IN, USA) were stopped due to abnormal liver biochemistry values flagged in 4 out of 45 patients during routine safety monitoring. Liver abnormalities were considered to be an off-target effect of that compound [23].

Interpretation of some of the analyses was limited by early termination of the ALZ2004 extension study and the small sample size, particularly the limited number of participants with normal cognition.

## Conclusions

In the 6-month ALZ2002 parent study, an atabecestat 50-mg dose was generally less well tolerated than lower doses with a higher frequency of participants discontinuing treatment early. Hepatic transaminase elevations were observed in the atabecestat treatment groups but not in the placebo group, resulting in dose reduction and discontinuation of the 50-mg dose. In addition, safety measures for monitoring hepatic function tests were implemented.

In the longer term extension study, some participants on active treatment in ALZ2004 double-blind and open-label periods exhibited transaminase elevations, including markedly abnormal ALT elevations accompanied by elevations in AST and in some cases by elevations in GGT. The elevations occurred between 3 and 12 months of treatment, and all resolved after discontinuation of study treatment except for one with a baseline elevation in transaminases. A trend for decreases from baseline in RBANS scores with 10 mg and 25 mg atabecestat treatment was observed in the extension study, indicating worsening of cognitive performance consistent with reports of other BACE inhibitors.

Given the frequency of hepatic enzyme elevations and concern for managing participant safety outside the structure of a clinical trial, dosing of atabecestat in ALZ2004 and EARLY was halted. The EARLY data, as well as data from multiple other BACE inhibitors, confirmed the trends for worsened cognition seen for the RBANS in the ALZ2002 and ALZ2004 studies. While BACE inhibition remains an intriguing mechanism to slow AD progression, a better understanding of BACE substrates is important prior to additional human studies.

## Supplementary information

**Additional file 1.** Supplementary information on the Methods.

**Additional file 2.** Supplementary Tables and Figures.

**Additional file 3.** Supplementary Material.

## Data Availability

The datasets analyzed during the present study are not publicly available, but they are available at http://yoda.yale.edu/johnson-johnson. Requests for access to the study data can be submitted through this independent site for review.

## Abbreviations

APP: Amyloid precursor protein
BACE1: β-Secretase1 enzyme
Aβ: Amyloid beta
AD: Alzheimer’s disease
CSF: Cerebrospinal fluid
MCI: Mild cognitive impairment
OL: Open-label
sAPP: Soluble amyloid precursor protein
t-tau: Total tau
p-tau_181_: Phosphorylated tau
CDR: Clinical Dementia Rating Scale
BMI: Body mass index
MRI: Magnetic resonance imaging
DSM: Diagnostic and Statistical Manual of Mental Disorders
PET: Positron emission tomography
ALT: Alanine aminotransferase
AST: Aspartate aminotransferase
GGT: Gamma-glutamyl transferase
ULN: Upper limit of normal
CDR-SB: CDR-sum of boxes
MMSE: Mini-Mental State Examination
RBANS: Repeatable Battery for the Assessment of Neuropsychological Status
CVLT-II: California Verbal Learning Test—second edition
CFI: Cognitive Function Index
ECL: Electrochemiluminescence
MSD: Meso Scale Discovery
PK: Pharmacokinetic
AUC_0–24 h_: Area under the plasma concentration-time curve at steady state
CL/F: Estimate of oral clearance
AEs: Adverse events
SOC: System Organ Class
TEAEs: Treatment-emergent adverse events
SAE: Serious adverse event
ARIA-E: Amyloid-related imaging abnormalities with edema or effusion
ARIA-H: ARIA with hemosiderin
C-SSRS: Columbia Suicide Severity Rating Scale
ANCOVA: Analysis of covariance
vMRI: Volumetric MRI
LS: Least squares
SD: Standard deviation
SE: Standard error
