# Supplementary material for: Long-term safety and tolerability of atabecestat (JNJ-54861911), an oral BACE1 inhibitor, in early Alzheimer’s disease spectrum patients: a randomized, double-blind, placebo-controlled study and a two-period extension study
Source: Alzheimers Res Ther. 2020 May 14;12:58. doi: 10.1186/s13195-020-00614-5 (PMC7227237; doi:10.1186/s13195-020-00614-5)
Supplement: Supplementary file 1 — Additional file 1. Supplementary information on the Methods. [file 13195_2020_614_MOESM1_ESM.docx]

**Additional File 1: Supplementary information on the Methods.**

**Methods**

**Clinical Dementia Rating Scale (CDR)**

The CDR assessed three domains of cognition (memory, orientation, judgment/problem solving) and three domains of function (community affairs, home/hobbies, personal care) using structured interviews of both the patient and a companion/informant by a trained rater and scored using a standard methodology. CDR global score for the six domains range from 0 to 3 with 0 indicating no dementia and 3 indicating severe dementia. It can be summed to obtain CDR Sum of Boxes (CDR-SB) with scores ranging from 0 to 18, a higher score indicates greater impairment. CDR global score has been used in AD trials as a global measure of disease progression.

**Repeatable Battery for the Assessment of Neuropsychological Status (RBANS)**

The RBANS is a battery of tests developed for cognitive assessment, detection and characterization of dementia, and is a widely used measure to differentiate healthy normal participants from those with early disease and AD patients and has been used in multinational AD clinical trials [1-3]. RBANS battery includes 12 subsets that measure 5 indices; the Digit Span and Coding subtests measure Attention, the Picture Naming and Semantic Fluency subtests measure Language, the Figure Copy and Line Orientation subtests measure Visuospatial/Construction, List Learning and Store Memory subtests measure Immediate Memory, and List Recall, List Recognition, Store Memory and Figure Recall measure Delayed Memory. The raw scores from the subsets were scaled together using an RBANS conversion table to create index scores that were summed to form the sum of index scores. The sum of index scores was converted to the total scale using the RBANS conversion table [1]. The lower the scores the more pronounced the impairment.

**California Verbal Learning Test-Second Edition (CVLT-II)**

The CVLT-II is a neuropsychological measure designed to quantify components of verbal learning, retention and retrieval has a large normative database with computerized scoring (Psychological Corporation 2000, San Antonio, Texas, USA). Sixteen (16) words were read out face-to-face to the participants five times with the instruction to memorize as many words as possible each time. Each of the words belonged to one of four categories: thus, there were four fruits, four herbs and spices, etc. The memory subtests included immediate recall (the sum of all words recalled immediately following each presentation long delay recall (the sum of both free recall and semantically cued recall of the words after an interval of at least 20 minutes), and long-delay recognition. For immediate and long delayed recall, higher scores indicate better recall.

The CVLT-II is a neuropsychological measure designed to quantify components of verbal learning, retention and retrieval has a large normative database with computerized scoring (Psychological Corporation 2000, San Antonio, Texas, USA). Sixteen (16) words were read out face-to-face to the participants five times with the instruction to memorize as many words as possible each time. Each of the words belonged to one of four categories: thus, there were four fruits, four herbs and spices, etc. The participants were asked to recite the words they could recollect immediately following each presentation (immediate recall) and after an interval of at least 20 minutes (long delay recall), which was then followed by a 32-item recognition subtest (long-delay recognition). Scores were derived for recall types, and higher scores indicate better recall

**Mini Mental State Examination (MMSE)**

The MMSE is a brief, validated 30-point questionnaire used to screen for cognitive impairment. MMSE rates participants on orientation (total score 10), registration (total score 3), attention, calculation (total score 5), recall (total score 3) and language (total score 9). An overall total score is obtained by summing all the item scores and is the outcome of interest. The maximum total score is 30, and the lower the score the more pronounced the impairment.

**Cognitive Function Index (CFI)**

The CFI is a modified version of the Mail-in Cognitive Function Screening Instrument [5], a participant- and study partner-reported outcome measure developed by the Alzheimer’s Disease Cooperative Study (ADCS). This assessment included 15 questions that assessed participant’s perceived ability to perform high level functional tasks in daily-life and their sense of overall cognitive functional ability. The participants and their partner/informant independently rated the participant’s abilities. The item responses were converted to corresponding numeric scores. The ADCS-CFI Total score was calculated as the sum of questionnaire items ranging from 0 to 14. Higher scores indicate greater impairment.

**References**

1. Randolph, C., Tierney, M. C., Mohr, E., & Chase, T. N. The Repeatable Battery for the Assessment of Neuropsychological Status (RBANS): Preliminary Clinical Validity. J. Clin Exp Neuropsychol,1998 Jun; 20(3), 310-319.

2. Kotani, S., Sakaguchi, E., Warashina, S., Matsukawa, N., Ishikura, Y. et al. Dietary supplementation of arachidonic and docosahexaenoic acids improves cognitive dysfunction. Neurosci Res, 2006 Oct; 56(2), 159-164.

3. Karantzoulis S, Novitski J, Gold M, Randolph C. The Repeatable Battery for the Assessment of Neuropsychological Status (RBANS): Utility in Detection and Characterization of Mild Cognitive Impairment due to Alzheimer's Disease. Arch Clin Neuropsychol. 2013 Jul 17. PubMed PMID: 23867976.

4. Delis D, Kaplan E, Kramer J, Ober B. The California Verbal Learning Test-II, San Antonio, TX. The Psychological Corporation, 2000.

5. Walsh, S. P., Raman, R., Jones, K. B., Aisen, P. S., & Alzheimer's Disease Cooperative Study Group. (2006). ADCS prevention instrument project: the mail-in cognitive function screening instrument (MCFSI). *Alzheimer Disease & Associated Disorders*, *20*, S170-S178.
