# Supplementary material for: Long-term safety and tolerability of atabecestat (JNJ-54861911), an oral BACE1 inhibitor, in early Alzheimer’s disease spectrum patients: a randomized, double-blind, placebo-controlled study and a two-period extension study
Source: Alzheimers Res Ther. 2020 May 14;12:58. doi: 10.1186/s13195-020-00614-5 (PMC7227237; doi:10.1186/s13195-020-00614-5)
Supplement: Supplementary file 2 — Additional file 2. Supplementary Tables and Figures. [file 13195_2020_614_MOESM2_ESM.docx]

**Additional File 2: Supplementary Tables and Figures**

**Supplementary Tables**

**Supplementary Table S1. Summary statistics of atabecestat individual plasma exposure estimates (AUC_0-24h_) in combined studies ALZ2002 and ALZ1005 (population pharmacokinetic analysis)**

| **Atabecestat Dose (mg)** | **N** | **Plasma Steady-State AUC_0-24h_ (ng h/mL)** | | | |
| --- | --- | --- | --- | --- | --- |
|  |  | **Mean (SD)** | **Median** | **Minimum** | **Maximum** |
| 5 | 21 | 566 (153) | 544 | 359 | 860 |
| 10 | 36 | 1159 (302) | 1081 | 717 | 1719 |
| 25 | 19 | 3382 (1564) | 2998 | 1532 | 6560 |
| 50 | 38 | 6104 (2694) | 5577 | 3065 | 13120 |

AUC_0-24h_=area under the concentration-time profile during the dosing interval at steady-state (AUC from 0 to 24 hours post dosing)

**Supplementary Table S2. Comparison of RBANS Total Scale and MMSE Total Score at week 52 with placebo from ANCOVA analysis; adjusting for baseline score and baseline CDR status for ALZ2004 double-blind safety analysis set**

| **Comparison** | **Treatment Group in ALZ2004 double-Blind, Period 1** | | |
| --- | --- | --- | --- |
|  | **Placebo** | **Ata 10 mg** | **Ata 25 mg** |
| **^Analysis set: Double-Blind Safety, N^** | ^35^ | ^29^ | ^26^ |
| **^RBANS Total Scale^** |  |  |  |
| **^Baseline, N^** | ^33^ | ^28^ | ^26^ |
| **^Mean (SD)^** | ^77.8 (15.67)^ | ^72.4 (22.64)^ | ^80.5 (19.19)^ |
| **^Double-blind Week 52, N^** | ^28^ | ^24^ | ^17^ |
| **^Mean (SD)^** | ^79.7 (21.91)^ | ^67.5 (22.05)^ | ^78.9 (21.74)^ |
| **^ANCOVA^** |  |  |  |
| **^LS Mean (SE)^** | ^78.7 (2.06)^ | ^74.7 (1.98)^ | ^73.1 (2.47)^ |
| **^95% CI)^** | ^(74.58; 82.82)^ | ^(70.70; 78.61)^ | ^(68.16; 78.04)^ |
| **^Difference of LS Means (minus Placebo)^** | ^-^ | ^-4.05^ | ^-5.60^ |
| **^95% CI^** | ^-^ | ^(-9.59; 1.50)^ | ^(-11.44; 0.24)^ |
| **^p-value (minus Placebo)^** | ^-^ | ^0.1499^ | ^0.0600^ |
| **^MMSE Total Scale^** |  |  |  |
| **^Baseline, N^** | ^33^ | ^27^ | ^26^ |
| **^Mean (SD)^** | ^26.1 (2.85)^ | ^24.6 (4.44)^ | ^26.0 (2.44)^ |
| **^Double-blind Week 52, N^** | ^28^ | ^26^ | ^22^ |
| **^Mean (SD)^** | ^24.4 (5.12)^ | ^22.4 (6.28)^ | ^24.0 (4.91)^ |
| **^ANCOVA^** |  |  |  |
| **^LS Mean (SE)^** | ^23.8 (0.77)^ | ^24.3 (0.70)^ | ^24.4 (0.78)^ |
| **^95% CI)^** | ^(22.31; 25.36)^ | ^(22.87; 25.66)^ | ^(22.83; 25.95)^ |
| **^Difference of LS Means (minus Placebo)^** |  | ^0.43^ | ^0.55^ |
| **^95% CI^** |  | ^(-1.58; 2.44)^ | ^(-1.41; 2.52)^ |
| **^p-value (minus Placebo)^** |  | ^0.6699^ | ^0.5751^ |

Baseline for Period 1 is defined as the pre-dose baseline value from the preceding study ALZ2002.
LS Means are the means of Week 52 RBANS Total Scale adjusted for Baseline RBANS Total Scale and for Baseline CDR status.
LS Means are the means of Week 52 MMSE Total Score adjusted for Baseline MMSE Total Score and for Baseline CDR status.
LS Means from ANCOVA model with the Week 52 RBANS Total Scale as response and the baseline RBANS Total Scale, the baseline CDR status and the treatment group as explanatory variables
LS Means from ANCOVA model with the Week 52 MMSE Total Score as response and the baseline MMSE Total Score, the baseline CDR status and the treatment group as explanatory variables
P-values Unadjusted for Multiple Comparisons.

**Supplementary Table S3. Summary of CDR-SB total scores, CFI participant and CFI study partner total scores, and CVLT-II scores and change from baseline by treatment group for ALZ2004 double-blind period 1 (safety analysis set)**

| **^Cognitive Outcome^** |  |  |  |  | **^Change from Baseline^** | | | |
| --- | --- | --- | --- | --- | --- | --- | --- | --- |
|  | **^N^** | **^Mean (SD)^** | **^Range^** | **^Base Mean^** | **^N^** | **^Mean (SD)^** | **^Std. Error^** | **^Range^** |
| **^CDR-SB^** |  |  |  |  |  |  |  |  |
| **^Placebo^** |  |  |  |  |  |  |  |  |
| **^Baseline^** | ^33^ | ^1.45 (1.195)^ | ^(0.0, 4.0)^ |  |  |  |  |  |
| **^Double-Blind Week 52^** | ^27^ | ^2.43 (2.340)^ | ^(0.0, 7.0)^ | ^1.28^ | ^25^ | ^1.34 (1.694)^ | ^0.339^ | ^(-0.5, 6.0)^ |
| **^Ata 10 mg^** |  |  |  |  |  |  |  |  |
| **^Baseline^** | ^28^ | ^1.61 (1.322)^ | ^(0.0, 3.5)^ |  |  |  |  |  |
| **^Double-Blind Week 52^** | ^26^ | ^2.85 (2.694)^ | ^(0.0, 12.0)^ | ^1.58^ | ^26^ | ^1.27 (2.051)^ | ^0.402^ | ^(-1.0; 8.5)^ |
| **^Ata 25 mg^** |  |  |  |  |  |  |  |  |
| **^Baseline^** | ^26^ | ^1.35 (0.903)^ | ^(0.0, 3.0)^ |  |  |  |  |  |
| **^Double-Blind Week 52^** | ^22^ | ^2.36 (2.019)^ | ^(0.0, 9.0)^ | ^1.41^ | ^22^ | ^0.95 (1.487)^ | ^0.317^ | ^(-1.0, 8.5)^ |
| **^CFI Participant Total Score^** |  |  |  |  |  |  |  |  |
| **^Placebo, Preclinical AD^** |  |  |  |  |  |  |  |  |
| **^Baseline^** | ^6^ | ^2.833 (1.9664)^ | ^(1.00, 6.50)^ |  |  |  |  |  |
| **^Double-Blind Week 52^** | ^6^ | ^2.417 (2.3541)^ | ^(0.50, 7.00)^ | ^2.833^ | ^6^ | ^-0.417 (1.1583)^ | ^0.4729^ | ^(-2.0, 1.0)^ |
| **^Placebo, MCI due to AD^** |  |  |  |  |  |  |  |  |
| **^Baseline^** | ^24^ | ^4.688 (3.5165)^ | ^(0.00, 13.50)^ |  |  |  |  |  |
| **^Double-Blind Week 52^** | ^23^ | ^4.217 (3.2257)^ | ^(0.00, 10.00)^ | ^4.579^ | ^19^ | ^-0.658 (2.9910)^ | ^0.6862^ | ^(-7.5, 4.0)^ |
| **^Ata 10 mg, Preclinical AD^** |  |  |  |  |  |  |  |  |
| **^Baseline^** | ^8^ | ^3.688 (3.2726)^ | ^(0.00, 10.00)^ |  |  |  |  |  |
| **^Double-Blind Week 52^** | ^8^ | ^3.063 92.4704)^ | ^(0.50, 7.50)^ | ^3.688^ | ^8^ | ^-0.625 (1.5755)^ | ^0.5570^ | ^(-2.5, 2.0)^ |
| **^Ata 10 mg, MCI due to AD^** |  |  |  |  |  |  |  |  |
| **^Baseline^** | ^18^ | ^5.111 (2.3674)^ | ^(0.50, 8.50)^ |  |  |  |  |  |
| **^Double-Blind Week 52^** | ^17^ | ^3.971 (2.6426)^ | ^(0.00, 8.50)^ | ^4.786^ | ^14^ | ^-0.643 (2.7205)^ | ^0.7271^ | ^(-6.0, 3.5)^ |
| **^Ata 25 mg, Preclinical AD^** |  |  |  |  |  |  |  |  |
| **^Baseline^** | ^6^ | ^2.083 (2.1775)^ | ^(1.00, 6.50)^ |  |  |  |  |  |
| **^Double-Blind Week 52^** | ^5^ | ^3.300 (1.7889)^ | ^(1.50, 6.00)^ | ^2.300^ | ^5^ | ^1.000 (1.1180)^ | ^0.5000^ | ^(-0.5, 2.5)^ |
| **^Ata 25 mg, MCI due to AD^** |  |  |  |  |  |  |  |  |
| **^Baseline^** | ^19^ | ^5.026 (3.6986)^ | ^(0.00, 12.00)^ |  |  |  |  |  |
| **^Double-Blind Week 52^** | ^17^ | ^5.500 (3.5089)^ | ^(0.50, 13.00)^ | ^5.563^ | ^16^ | ^-0.281 (3.3064)^ | ^0.8266^ | ^(-6.0, 5.0)^ |
| **^CFI Partner Total Score^** |  |  |  |  |  |  |  |  |
| **^Placebo, Preclinical AD^** |  |  |  |  |  |  |  |  |
| **^Double-Blind Week 52^** | ^6^ | ^1.500 (1.6733)^ | ^(0.00, 4.00)^ | ^1.667^ | ^6^ | ^-0.167 (1.6330)^ | ^0.6667^ | ^(-2.5, 2.5)^ |
| **^Placebo, MCI due to AD^** |  |  |  |  |  |  |  |  |
| **^Double-Blind Week 52^** | ^22^ | ^5.447 (3.8378)^ | ^(0.00, 13.00)^ | ^4.139^ | ^18^ | ^1.296 (2.7603)^ | ^0.6506^ | ^(-3.0, 7.0)^ |
| **^Ata 10 mg, Preclinical AD^** |  |  |  |  |  |  |  |  |
| **^Double-Blind Week 52^** | ^8^ | ^2.875 (2.6559)^ | ^(0.00, 7.50)^ | ^3.000^ | ^8^ | ^-0.125 (0.9161)^ | ^0.3239^ | ^(-1.0, 1.5)^ |
| **^Ata 10 mg, MCI due to AD^** |  |  |  |  |  |  |  |  |
| **^Double-Blind Week 52^** | ^18^ | ^5.778 (3.4137)^ | ^(0.00, 10.50)^ | ^4.929^ | ^14^ | ^0.571 (2.3847)^ | ^0.6373^ | ^(-4.0, 4.0)^ |
| **^Ata 25 mg, Preclinical AD^** |  |  |  |  |  |  |  |  |
| **^Double-Blind Week 52^** | ^5^ | ^3.500 (2.1213)^ | ^(0.00, 5.00)^ | ^2.300^ | ^5^ | ^1.200 (3.5461)^ | ^1.5859^ | ^(-4.5, 4.0)^ |
| **^Ata 25 mg, MCI due to AD^** |  |  |  |  |  |  |  |  |
| **^Double-Blind Week 52^** | ^17^ | ^5.966 (3.8180)^ | ^(0.00, 12.50)^ | ^4.094^ | ^16^ | ^1.651 (2.7814)^ | ^0.6954^ | ^(-2.5, 9.5)^ |
| **^CVLT-II Score, Long Delay Recall^** |  |  |  |  |  |  |  |  |
| **^Placebo, Preclinical AD^** |  |  |  |  |  |  |  |  |
| **^Baseline^** | ^6^ | ^19.5 (8.38)^ | ^(9; 30)^ |  |  |  |  |  |
| **^Double-Blind Week 12^** | ^6^ | ^22.0 (8.29)^ | ^(8; 31)^ | ^19.5^ | ^6^ | ^2.5 (3.45)^ | ^1.41^ | ^(-1; 7)^ |
| **^Placebo, MCI due to AD^** |  |  |  |  |  |  |  |  |
| **^Baseline^** | ^25^ | ^9.9 (8.72)^ | ^(1; 32)^ |  |  |  |  |  |
| **^Double-Blind Week 12^** | ^26^ | ^11.1 (9.85)^ | ^(0; 32)^ | ^10.1^ | ^23^ | ^0.1 (4.0)^ | ^0.83^ | ^(-11; 5)^ |
| **^Ata 10 mg, Preclinical AD^** |  |  |  |  |  |  |  |  |
| **^Baseline^** | ^8^ | ^15.8 (12.29)^ | ^(1; 30)^ |  |  |  |  |  |
| **^Double-Blind Week 12^** | ^8^ | ^17.5 (11.63)^ | ^(3; 30)^ | ^15.8^ | ^8^ | ^1.8 (1.75)^ | ^0.62^ | ^(0; 5)^ |
| **^Ata 10 mg, MCI due to AD^** |  |  |  |  |  |  |  |  |
| **^Baseline^** | ^20^ | ^8.9 (8.03)^ | ^(1; 30)^ |  |  |  |  |  |
| **^Double-Blind Week 12^** | ^20^ | ^9.4 (9.03)^ | ^(1; 29)^ | ^8.8^ | ^19^ | ^-0.4 (3.27)^ | ^0.75^ | ^(-7; 8)^ |
| **^Ata 25 mg, Preclinical AD^** |  |  |  |  |  |  |  |  |
| **^Baseline^** | ^6^ | ^22.5 (9.67)^ | ^(5; 31)^ |  |  |  |  |  |
| **^Double-Blind Week 12^** | ^6^ | ^23.2 (11.16)^ | ^(4; 32)^ | ^22.5^ | ^6^ | ^0.7 (3.01)^ | ^1.23^ | ^(-4; 5)^ |
| **^Ata 25 mg, MCI due to AD^** |  |  |  |  |  |  |  |  |
| **^Baseline^** | ^19^ | ^9.4 (6.81)^ | ^(2; 28)^ |  |  |  |  |  |
| **^Double-Blind Week 12^** | ^19^ | ^9.5 (6.18)^ | ^(0; 25)^ | ^9.5^ | ^18^ | ^-0.2 (3.37)^ | ^0.79^ | ^(-6; 5)^ |

Notes: Baseline for Period 1 is defined as the pre-dose baseline value from the preceding parent study ALZ2002.
CDR-SB = Clinical Dementia Rating Scale Sum of Boxes total score calculated as the sum of the ratings from 6 domains (cognition:memory, orientation, judgment/problem solving; function:community affairs, home/hobbies, personal care) with higher scores indicating greater impairment,
CFI= Cognitive function index, higher scores indicate greater impairment
CVLT-II= California verbal learning test, second edition. Higher scores on long delay recall indicate better performance

**Supplementary Figures**

**Supplementary Figure 1. (A) Box-whisker plots of percent change from baseline for plasma Aβ_1-40_ biomarker level by final dose groups at end of Month 6 of atabecestat treatment in ALZ2002early AD population. (B) Percent change from baseline time-profile for plasma Aβ_1-40_ levels to 52 weeks in ALZ2004 double-blind period.**

A) Percent change in plasma Aβ_1-40_ level by ALZ2002 treatment groups at end of Month 6*

**
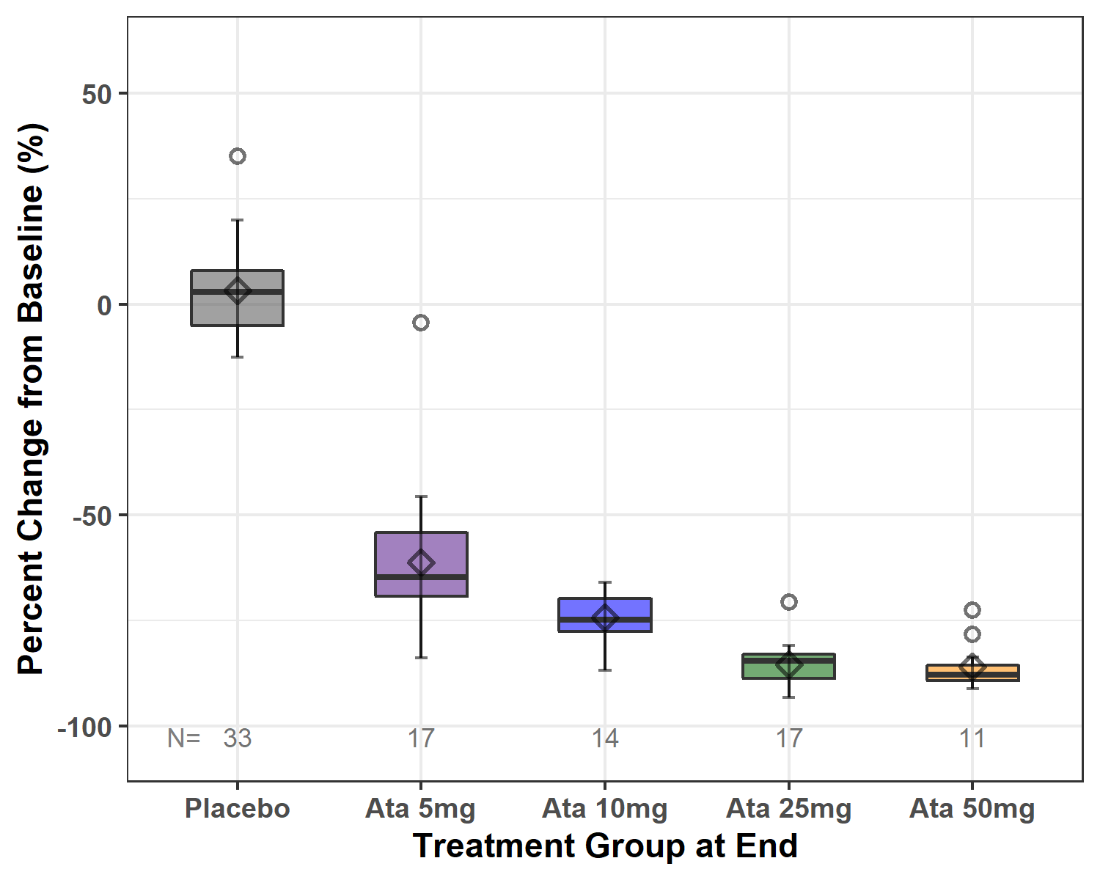
**

***** Outlier values of 153% and 550% (Placebo) and 122%, 350%, and 381% (Ata 5mg) not shown**.**

B) Percent change in plasma Aβ_1-40_ level by ALZ2004 double-blind period treatment groups

**
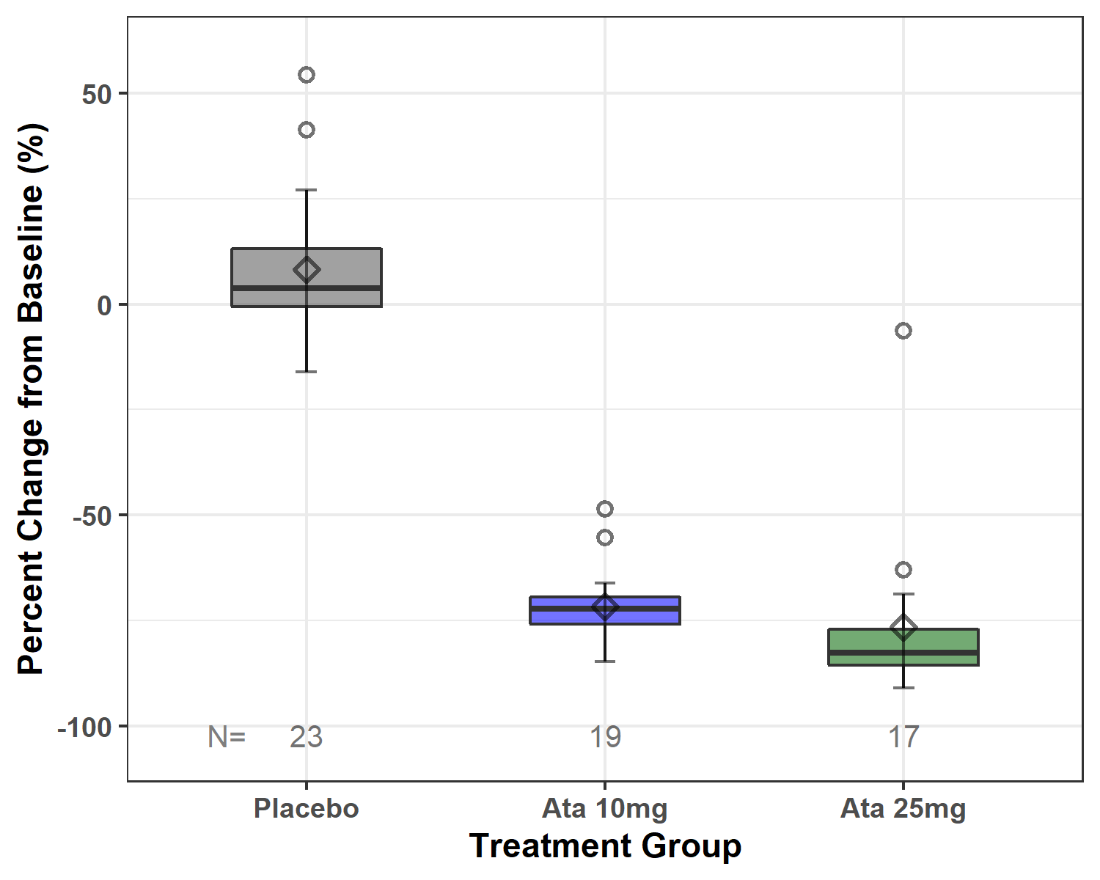
**
