# Supplementary material for: Long-term safety and tolerability of atabecestat (JNJ-54861911), an oral BACE1 inhibitor, in early Alzheimer’s disease spectrum patients: a randomized, double-blind, placebo-controlled study and a two-period extension study
Source: Alzheimers Res Ther. 2020 May 14;12:58. doi: 10.1186/s13195-020-00614-5 (PMC7227237; doi:10.1186/s13195-020-00614-5)
Supplement: Supplementary file 3 — Additional file 3. Supplementary Material. [file 13195_2020_614_MOESM3_ESM.docx]

**Supplementary Material**

The supplementary material has been provided by the authors to give readers additional information about the study and results.

Supplement to: Gerald Novak, Johannes Rolf Streffer, Maarten Timmers, David Henley et al. Long-term safety and tolerability of Atabecestat (JNJ-54861911), an oral BACE1 inhibitor in early Alzheimer's disease spectrum patients: Randomized double-blind placebo-controlled and open-label extension studies.
